# Isolation, characterization, identification, genomics and analyses of bioaccumulation and biosorption potential of two arsenic-resistant bacteria obtained from natural environments

**DOI:** 10.1038/s41598-024-56082-6

**Published:** 2024-03-08

**Authors:** Vivek Roy, Barnan Kumar Saha, Samarpita Adhikary, Madhumita G. Chaki, Monalisha Sarkar, Ayon Pal

**Affiliations:** Microbiology and Computational Biology Laboratory, Department of Botany, Raiganj University, Raiganj, West Bengal 733134 India

**Keywords:** Environmental microbiology, Soil microbiology, Water microbiology, Microbiology, Environmental sciences

## Abstract

Arsenic (As) is a significant contaminant whose unrestrained entrance into different ecosystems has created global concern. At the cellular level, As forms unsteady intermediates with genetic materials and perturbs different metabolic processes and proper folding of proteins. This study was the first in this region to explore, isolate, screen systematically, and intensively characterize potent As-tolerant bacterial strains from natural environments near Raiganj town of Uttar Dinajpur, West Bengal. In this study, two potent Gram-negative bacterial strains with high tolerance to the poisonous form of As, i.e., As(III) and As(V), were obtained. Both the isolates were identified using biochemical tests and 16S rRNA gene sequencing. These bacteria oxidized toxic As(III) into less poisonous As(V) and depicted tolerance towards other heavy metals. Comparative metabolic profiling of the isolates in control and As-exposed conditions through Fourier-transform infrared spectroscopy showed metabolic adjustments to cope with As toxicity. The metal removal efficiency of the isolates at different pH showed that one of the isolates, KG1D, could remove As efficiently irrespective of changes in the media pH. In contrast, the efficiency of metal removal by PF14 was largely pH-dependent. The cell mass of both the isolates was also found to favourably adsorb As(III). Whole genome sequence analysis of the isolates depicted the presence of the *arsRBC* genes of the arsenic operon conferring resistance to As. Owing to their As(III) oxidizing potential, high As bioaccumulation, and tolerance to other heavy metals, these bacteria could be used to bioremediate and reclaim As-contaminated sites.

## Introduction

Arsenic (As), a metalloid, is widely distributed in the lithosphere, atmosphere, and hydrosphere^1^. It is heavily toxic owing to its various structural forms, oxidative-reductive states, affinity towards water, absorption rate, and elimination^2^. It commonly associates with chloride, sulphur, and oxygen^2^ to generate various inorganic arsenicals and binds with hydrogen and carbon to produce organic arsenicals^1^. This heavy metal is found in the biosphere as arsine gas (− 3), elemental As (0), arsenite (+ 3), and arsenate (+ 5) form^3^. Out of the four, inorganic arsenite [As(III)] and arsenate [As(V)] are the most common in nature, notorious for their toxicity. Trivalent inorganic arsenite is 70 times more poisonous than organic variants of As, and ten times more than pentavalent arsenate^4^. Both soil and water are considered common sites for the origin of As contamination^5^. The sources for this wide distribution of As are both geogenic and anthropogenic. Human-induced sources for introducing As into the environment include the use of organic and inorganic pesticides; preservatives^2^; mining; ore smelting like copper, lead, gold, and many more where secondary by-products rich in As are generated; various large-scale industries that use As as an alloy^6^; combustion of non-renewable sources of energy, As based fertilizers and manures^1^.

One of the primary modes for the worldwide distribution of As is water. Surface and sub-surface water contamination by species of As has already been reported in many countries around the globe^4^. The water commonly used in irrigation is sourced from groundwater polluted with As, which results in poor crop growth, low yields, and accumulation in various body parts of these crops. The ultimate consequences are bioaccumulation at the trophic level by gaining access through the food chain^7^. According to the US Environmental Protection Agency, any form of As could give rise to significant health issues in humans or livestock. Various agencies have already enlisted As as a “Group 1” or “Class A” carcinogen^6^; some have set it as “rank one” among elements due to its higher affinity towards lipids and tendency to deposit in fat tissues^8^. The chronic consumption of As by humans leads to numerous cancerous phenotypes in various vital organs^4,9^ and non-carcinogenic side-effects^10^; and thus is referred to as the king of poisons due to its carcinogenic, tumorogenic and mutagenic properties^2^. At the cellular level, from microbes to animals, As(V) forms unsteady intermediates by replacing inorganic phosphate from proteins and genetic materials. As(V) also hampers the generation of ATP, ATP-driven metabolic processes, transportation, signal transduction, etc. Like As(V), As(III) toxicity is also quite rampant. As(III) has high affinities towards sulfhydryl and thiol groups in various biomolecules, and upon binding with these biomolecules it inhibits or disrupts different metabolic pathways such as biosynthesis of deoxyribonucleotides, nucleic acid repair system, folding of proteins etc^11^.

Microorganisms are significant contributors to various ecological phenomena like nutrient cycling and detoxification of toxic elements^12^. The detoxification of harmful compounds or elements by bacteria depends on their cellular physiology and metabolism. A number of bacteria like *Agrobacterium tumefaciens* GW4, *Rhizobium* sp. NT-26 have been reported to utilize these xenobiotics (like As) as their source of energy or to sustain their growth^13^. Though As is highly poisonous to humans and other animals, As-tolerant bacteria can reduce As toxicity significantly. Such bacteria have many intrinsic mechanisms to scavenge As from their surrounding environment, like methylation, demethylation, complex formation, biosorption, oxido-reduction reactions, and others^14^. Some bacteria also code for a unique protein, arsenite oxidase, which can oxidize As(III) into its less potent form, As(V)^3^. Such bacteria showing tolerance towards As and biotransformation ability to reduce the deleterious effect of As are well characterized and usually have been isolated from contaminated areas. Still, only a few bacteria with such potentiality have been isolated from uncontaminated regions^15^.

Considering the toxicity, the multitude of effects on human health, and the absence of viable documentation on As-tolerant bacteria in and around Raiganj, this study aimed to isolate, characterize, and identify indigenous As-tolerant bacteria present in soil and river water samples from Kulik River and Kulik Forest in Raiganj. The bacterial strains thoroughly analyzed during this study were specifically selected based on their tolerance to elevated concentrations of As, i.e., As(III) and As(V). Scanning electron microscopy (SEM) was utilized to study bacterial morphology, and using Fourier-transform infrared spectrometry (FTIR), metabolic profiling was done to identify the probable functional groups expressed by the bacterial cell during their exposure to toxic As(III). The growth kinetics of the As-tolerant isolates were studied in the absence and presence of As stress. The As(III) bioaccumulation and biosorption ability of the potent indigenous bacterial isolates along with their As(III) biotransformation ability, were also analyzed. Whole genome sequencing of the tolerant isolates was performed to determine the genes responsible for conferring resistance to As. The indigenous As-tolerant bacteria analyzed in this study are the first to be reported from the Uttar Dinajpur region of West Bengal, India. The microbial biodiversity of this region is largely unexplored, and the detection of potent As-tolerant bacteria from this region will pave the way for more research in this unexplored area. It can help counter severe As contamination predominant in the neighbouring districts of Malda and Murshidabad^16,17^.

## Results and discussion

### Isolation and minimum inhibitory concentration (MIC) determination of As-tolerant bacterial isolates

To estimate the degree of susceptibility of the obtained bacterial isolates towards As, MIC was calculated using two different forms of inorganic As, i.e., arsenite [As(III)] and arsenate [As(V)]. These two forms of As are usually found in nature and are more toxic than the others. The MIC here is defined as the lowest concentration of the metalloid (As) in which growth is absent even after 72 h of incubation at 35 °C. Studies of MIC are crucial as they lay a path to deciphering the importance of indigenous bacteria in As treatment biologically^12^.

In this study, 18 bacterial isolates demonstrating preliminary tolerance to As(III) and As(V) were obtained. The details of these isolates are given in the supplementary Table S1. Two bacterial isolates designated as KG1D and PF14 showed relatively elevated MIC against As. The colony morphology of the two bacterial strains is depicted in Fig. S1. Both bacterial strains were found to grow profusely in both forms of As. The MIC of PF14 was 500 and 2500 µg mL^-1^ for the trivalent and pentavalent inorganoarsenals, respectively. In comparison, KG1D showed a MIC value of 600 and 1800 µg mL^−1^ for As(III) and As(V), respectively. As-tolerant bacteria have been reported from various regions across the globe, such as polluted water from an Argentinian village well^18^, sediments from the Vietnam Sea coast^19^, metal-laden soil of Portugal^20^, spring water in an Iranian province^21^, uranium mines in India^22^, silver and gold mines of the Korean Republic^23^ and Poland^24^. All this indicates that the presence of As-tolerant bacteria in nature is worldwide**,** and the tolerance determinants or different physiological mechanisms to cope with As stress might have evolved in the pre-historic era^25^. This tolerance towards arsenical compounds in bacteria and prokaryotes is mediated by resistant traits retained within chromosomes or could be extra-chromosomal in origin^26^.

Bacteria with varying degrees of tolerance have been reported from arsenic-polluted environmental sites, including Gram-negative bacteria from industrial effluents with a MIC value of 370 µg mL^−1^ for As(III)^27^. Another study reported Gram-positive and negative rhizobacteria with MIC of 1500 µg mL^−1^ for As(V)^14^. Six Gram-positive bacteria have been reported from a contaminated lake that can tolerate up to 23.97 mg mL^−1^ and 1.198 mg mL^−1^ of As(V) and As(III)^28^, respectively.

As content of the soil and water samples (supplementary Table S2) from which the isolates were obtained in this study did not show any significant trace of As (< 0.01 ppm), indicating the absence of As contamination. The pH of the isolation site was also found to be neutral or near neutral. Although isolated from As uncontaminated sites, these strains depicted a high degree of tolerance towards the toxic form of As. While many bacteria have been reported primarily from As contaminated sites, very little is known about indigenous isolates from uncontaminated sites.

### Determination of minimum biocidal concentration (MBC)

MBC for any heavy metal is the minimum concentration lethal for the test microorganism^29^. In the case of PF14, after transferring the bacteria to a fresh minimal salts medium (MSM), the lack of turbidity after 48 h indicated the absence of viable cell mass. In As(III), growth of PF14 was lacking at 6000 µg mL^−1^, and this lethal concentration was regarded as its MBC value. But for KG1D, the MBC value (15,000 µg mL^−1^) against As(III) was much higher than PF14.

### Tolerance to other heavy metals and MIC estimation

The tolerance of the selected bacterial strains to other heavy metals was determined by culturing them in MSM supplemented with various heavy metals at different concentrations. As these bacterial strains are environmental isolates, it is evident that these isolates would have different tolerance levels to other heavy metals, and the same was observed in this study. The selected isolates showed a wide range of tolerance towards various heavy metals in addition to As(III) and As(V). For PF14, the MICs for Cd, Cu, Cr, Ni, Zn and Hg were 200, 100, 300, 100, 300 and 5 µg mL^−1^, respectively. On the contrary, KG1D showed MICs of 175, 100, 100,100, 1000 and 10 µg mL^−1^ for those same heavy metals, respectively (Table 1). It is not unknown that resistance towards such heavy metals is provided by intrinsic factors harboured by most strains of bacteria discovered from marine, freshwater, polluted or unpolluted soil, effluents, etc. Diverse protective mechanisms have evolved in various species of bacteria to adapt to stressed environments. The most commonly used detoxifying mechanisms include extracellular barriers, removal of metals via efflux pumps, entrapment within the cell, extracellular metal trapping via secretion of various chelating agents, and enzymatic alteration to less toxic form^30^.

**Table 1:** The minimum inhibitory concentrations (µg mL^−1^) of different heavy metals of the tolerant bacterial isolates KG1D and PF14.

Name of the isolate	Cd	Cu	Cr	Ni	Zn	Hg
KG1D	175	100	100	100	1000	10
PF14	200	100	300	100	300	5

### Morphological and biochemical characterization of the selected isolates

The colony morphology appeared circular and smooth when KG1D and PF14 were grown in culture plates. Isolate KG1D showed brick red colonies, while the colonies of PF14 were greyish. Gram staining revealed that both the isolates KG1D and PF14 were rod-shaped Gram-negative bacteria. The cellular morphology observed under SEM for the isolates in control as well as in the presence of As(III) is provided in supplementary Fig. S2. The SEM of KG1D and PF14 in the absence of As(III) showed that they exist either as aggregates of loosely packed bacilli or as single cells. In presence of As(III), the formation of relatively smaller cellular aggregates was found to be predominant in both the isolates. The morphology of KG1D cells was found to be relatively less impacted in presence of As(III) compared to PF14. The clear distinction in the biochemical profiles of both isolates provided an initial indication of their distinct taxonomic position based on Bergey’s Manual of Systematic Bacteriology 2nd edition. Both isolates responded negatively to the indole test. In addition, KG1D showed a negative response towards starch hydrolysis, urease, and oxidase tests, but PF14 responded positively towards these three tests. Like KG1D, PF14 exhibited a negative reaction toward the Voges-Proskauer’s test, but unlike PF14, KG1D showed a positive response to the methyl-red test. Both the isolates successfully utilized citrate as the sole source of carbon. The isolate PF14 failed to liquefy gelatin in contrast to KG1D. The results of the biochemical tests have been summarized in supplementary Table S3.

### Molecular identification of As-resistant strains

Based on the BLAST results of 16S rRNA gene sequences, the isolate KG1D exhibited 99.56% identity with the type strain *Serratia marcescens* NBRC 102204^T^. In comparison, PF14 demonstrated 98.56% identity with the type strain *Alcaligenes faecalis* subsp. *phenolicus* DSM 16503^T^. *Serratia* belongs to the family Enterobacteriaceae of the order Enterobacterales, while *Alcaligenes* is a member of the family Alcaligenaceae under the order Burkholderiales. Both strains are members of the phylum Pseudomonadota. The GenBank accession numbers for the 16S rRNA gene sequences of strain KG1D and PF14 are OQ825954 and OR198906, respectively.

### Growth kinetics

**Figure 1:**
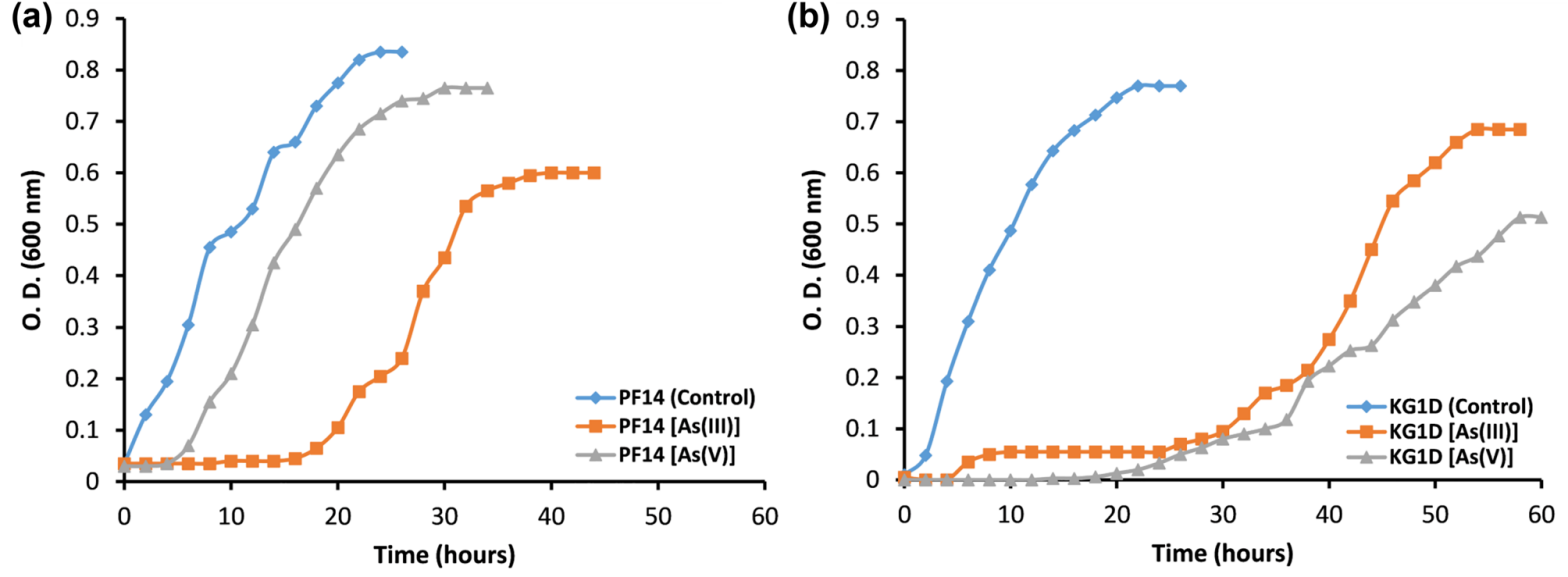
Growth curve of the isolates (**a**) PF14 and (**b**) KG1D in both control (metal-devoid) growth medium as well as in As(III) and As(V) supplemented medium at their respective MTCs at 35 °C. The MTC of As(III) for KGID and PF14 are 500 and 400 µg mL^−1^, respectively, while the MTC of As(V) are 1700 and 2400 µg mL^−1^ for KG1D and PF14, respectively.

The growth curves of both the strains KG1D and PF14 supplemented with varying concentrations of As(III) and As(V) are depicted in Fig. 1. The two bacteria grew profusely with a negligible lag phase when cultured in As-free conditions. The growth rate of PF14 and KG1D in control (without As) was 3.79 and 4.02 generations h^-1^, respectively. In the presence of As(V), the lag phase extended to 4 h and 30 h for PF14 and KG1D, respectively. The growth rate of PF14 and KG1D was also reduced to 2.29 and 2.00 generations h^−1^, respectively, in the presence of As(V). The lag phase was extended to 18 h and 26 h for PF14 and KG1D, respectively when As(III) was present in the culture media. The growth rate in this scenario declined further and reached 1.06 and 0.76 generations h^−1^ for PF14 and KG1D, respectively. These observations depicted the relatively increased inhibitory effects of As(III) on the growth of both isolates compared to As(V). Since As is known to disrupt metabolic activities^11^, affected cells require increased time to adjust and achieve their active growth phase. Isolate PF14 (Fig. 1a) depicted a relatively shorter lag phase than KG1D (Fig. 1b) in the presence of both forms of As as stressors. Compared to KG1D, PF14 demonstrated a relatively higher growth rate in control and both forms of As. The comparative growth rate and generation time of KG1D and PF14 in different conditions are shown in Table 2.

**Table 2:** The growth rate (generations h^−1^) and generation time (min) of the isolates in control and As-supplemented media.

Growth conditions	Growth rate (generations h^-1^)		Generation time (min)	
	KG1D	PF14	KG1D	PF14
Positive control (without As)	4.02	3.79	14.94	15.85
As(V) supplemented media	2.00	2.29	30.00	26.16
As(III) supplemented media	0.76	1.06	79.44	56.54

### Effect of pH and As on the bacterial strains

The effect of pH and the combined effect of both pH and As(III) on the growth of the isolates was studied, and the results are shown in supplementary Figs. S3 and S4, respectively. Without As (positive control), the bacterial strains KG1D and PF14 demonstrated maximum pH tolerance up to 11 but thrived best between pH 5 and 8. An increase in pH above 8 leads to a continuous decline in growth. In the presence of As(III), the favourable pH for growth was 7 for both strains. Previous studies have reported that pH 7 is optimal for both the growth and uptake of heavy metals in metal-stressed conditions^31^. However, isolate KG1D flourished quite well in a medium supplemented with As(III), even at pH 9. Both the strains showed moderate growth at pH 5 but lacked growth completely at pH 4 and 11. However, compared to PF14, the growth of KG1D at pH 5 was still high. These observations reflect that the bacterium KG1D thrives relatively better when both pH and As stress exist simultaneously in the culture medium.

The threshold pH for optimal growth differs from species to species, and unsuitable pH adversely affects bacterial growth^32^. pH profoundly affects microbes by altering their metabolism and thus influences the metal removal efficiency^33^. The environmental pH affects the overall surface charge of bacteria, thus reducing its ability to remove heavy metal ions. Studies have reported that with the increase in pH, the heavy metal uptake capacity of microorganisms increases but within a specific limit. Beyond this limit, the removal rate starts to decrease^34^. With the gradual increase in pH (metal ion concentration being constant at 300 µg mL^−1^), there was a gradual decline in bacterial growth. At low pH, the anionic functional groups on the cell surface get protonated rapidly, and this positive charge repels the positive metal ions. Protons even compete with the cationic metal for the binding sites in bacterial cells, thus hindering the interaction of metal ions and ultimately leading to a decline in binding and uptake^35^. When the pH increases, more binding sites with negative charges get exposed and bind with cationic metal ions, thus increasing metal removal. But with a further rise in pH, metals precipitate in the form of hydroxides, which are less soluble at higher pH, resulting in lower adsorption ability^36^. When taken inside the cell, As is modified via bacterial intracellular metabolic pathways, including methylation, redox reactions, and demethylation, to detoxify the cellular compartment or provide electrons for cell respiration. The bioaccumulation of metals inside cells after a specific threshold concentration gets inhibited, and metal ions are pumped out by efflux proteins^37^ to protect the cell from the detrimental effect of high concentration of metal ions in the cytoplasm^31^.

### As(III) oxidizing ability of the tolerant isolates

**Figure 2:**
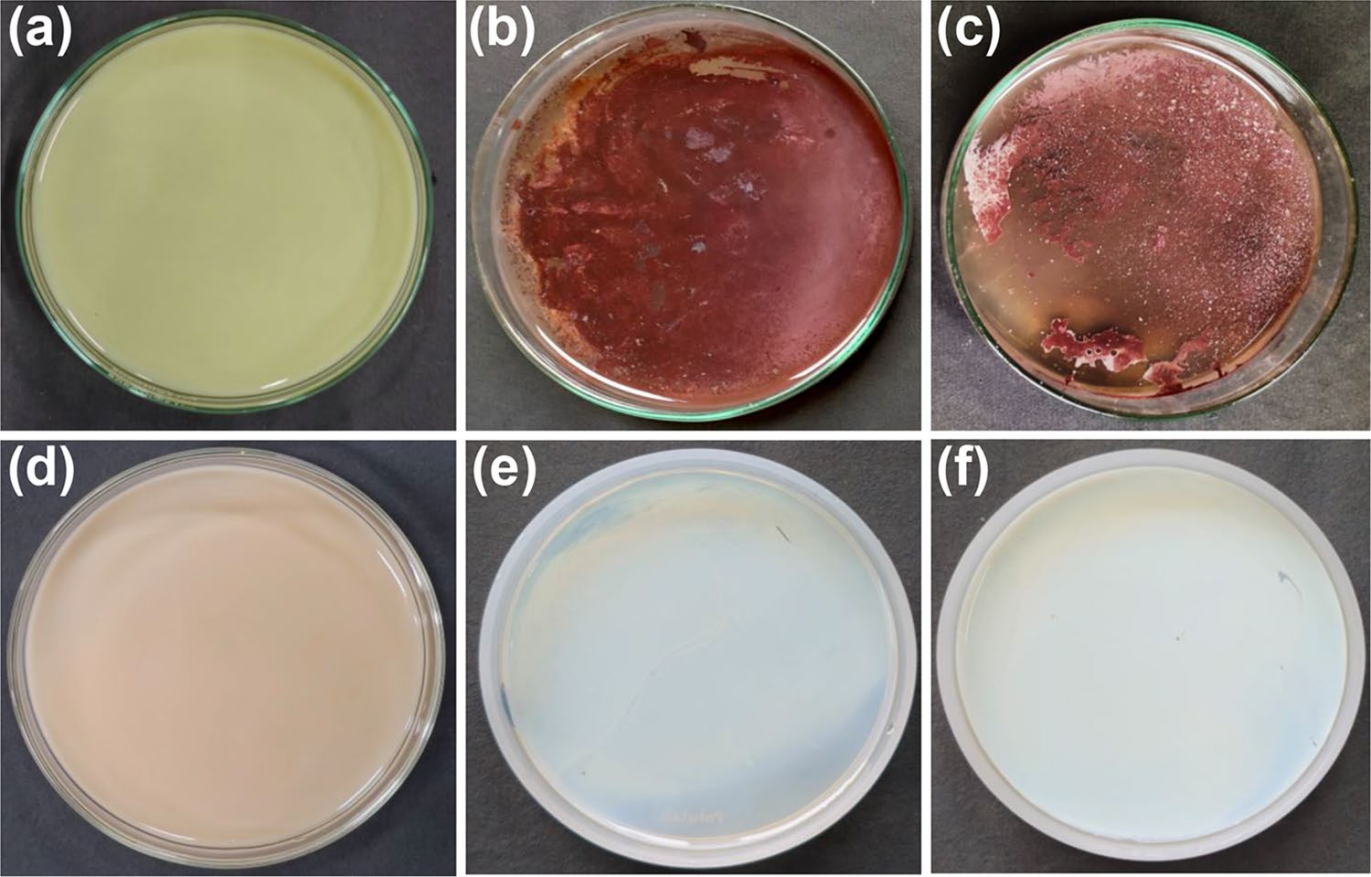
Bacteria-mediated As(III) oxidation in CDM agar medium detected using AgNO_3_ assay. Culture plate (**a**) depicts negative control with As(III), plates (**b**) and (**c**) shows KG1D and PF14, respectively grown in As(III). Culture plate (**d**) depicts negative control with As(V), while (**e**) and (**f**) depicts KG1D and PF14, respectively grown in presence of As(V). All the plates are treated with 0.1 M AgNO_3_ solution.

A qualitative silver nitrate (AgNO_3_) screening assay was utilized to determine the As(III) oxidizing nature of the tolerant isolates. The presence of a brownish precipitate in the vicinity of bacterial growth on solid media (Fig. 2) indicates the formation of silver arsenate (Ag_3_AsO_4_). It indicates the As(III) oxidizing ability of the bacteria. While KG1D demonstrated consistent arsenite oxidizing ability, PF14 was slightly inconsistent. As(III) oxidation was detected in all the culture plates inoculated with KG1D, while PF14 could oxidize As(III) in only 80% of the culture plates. The presence of silver arsenate reveals the ability of the isolate to produce arsenite oxidizing enzyme in the periplasm, and its expression takes place primarily in the presence of As(III). This oxidizing property helps bacteria detoxify the highly toxic form of As into its less toxic form. Bacteria with this inherent oxidation property generally use it either to produce energy for growth, as a protective mechanism, or both^25^. Bacteria have been reported from soil capable of oxidizing As(III) to extract energy both in the presence or absence of oxygen in the environment^38^. Though initially isolated from the oxidizer *Alcaligenes faecalis*, this oxidase enzyme is present in individuals belonging to different groups of bacteria^13^.

### Comparative analysis of As removal and bioaccumulation by the tolerant bacterial strains

**Figure 3:**
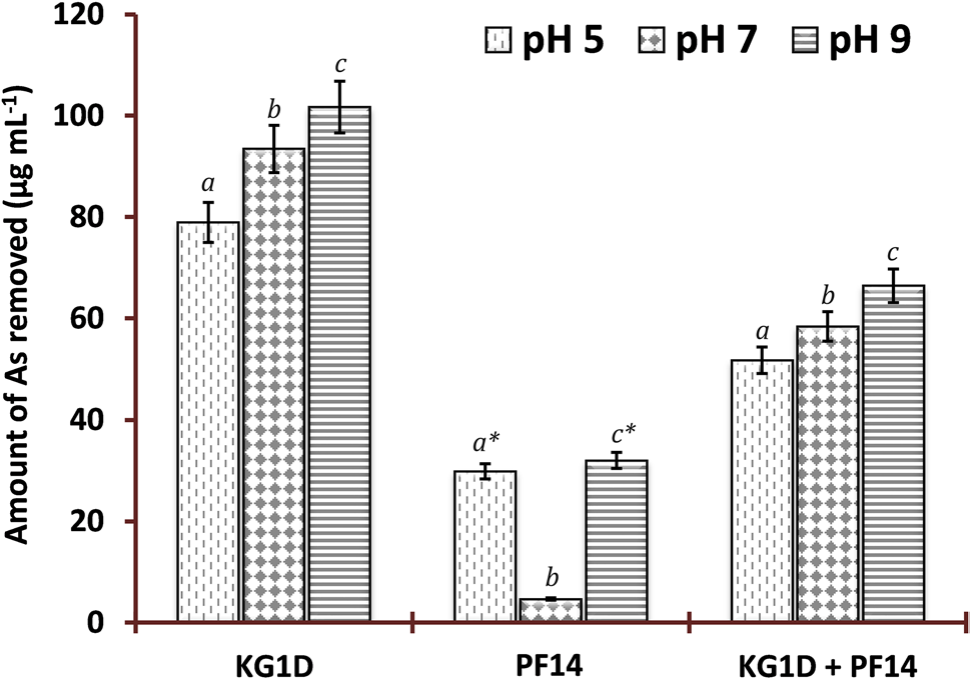
A comparative representation of As removal by the isolates, PF14 and KG1D, singly and cumulatively at different pH. Based on 1-ANOVA, the letter ‘*a*’ represents significant difference in As removal between groups at pH 5, ‘*b*’ represents significant difference between groups at pH 7, ‘*c*’ represents significant difference between groups at pH 9, and the symbol ‘*’ represents significant difference vs pH 7 within groups (*p* < 0.01).

The metal removal efficacy of the selected isolates from their environment (growth medium) was determined by culturing them separately and cumulatively at different pH. The obtained data was analyzed statistically by one-way analysis of variance (1-ANOVA) followed by Holm-Sidak post hoc tests to find out if there exists any significant difference in As removal capacity by the different isolates either singly or together in diverse pH. A graphical representation of the comparative As removal by both isolates individually or cumulatively at various pH is given in Fig. 3.

A 1-ANOVA using As removal data of PF14 showed that pH significantly affects the amount of As removed by the cells (*F* = 53.472, *df* = 2, *p* < 0.001). Pairwise multiple comparisons using the Holm-Sidak method showed that As removal by PF14 differs between pH 5 and 7 and between pH 7 and 9. No significant difference in removal of As by PF14 was observed between pH 5 and 9. The 1-ANOVA on As removal data by KG1D at different pH (5, 7 and 9) did not detect any statistically significant differences (*F* = 3.981 with *df* = 2*, p* = *0.058*) among the treatment groups. When both the isolates were used cumulatively for As removal at different pH (5, 7 and 9), no significant effect of pH on As removal was evident using 1-ANOVA (*F* = 1.579, *df* = 2, *p* = 0.258).

A 1-ANOVA on the comparative amount of As removed by the monoculture of PF14, KG1D, and mixed culture (PF14 + KG1D) at different pH (5, 7 and 9) was also performed to detect if there exists any significant difference in As removal by PF14, KG1D and PF14 + KG1D at different pH. A statistically significant difference was seen in the amount of As removed in the case of pH 5 (*F* = 29.473, *df* = 2, *p* < 0.001), pH 7 (*F* = 101.913, *df* = 2, *p* < 0.001), and pH 9 (*F* = 46.189, *df* = 2, *p* < 0.001). Pairwise multiple comparisons using the Holm-Sidak test detected significant differences at *p* < 0.01 level in all the instances.

**Figure 4:**
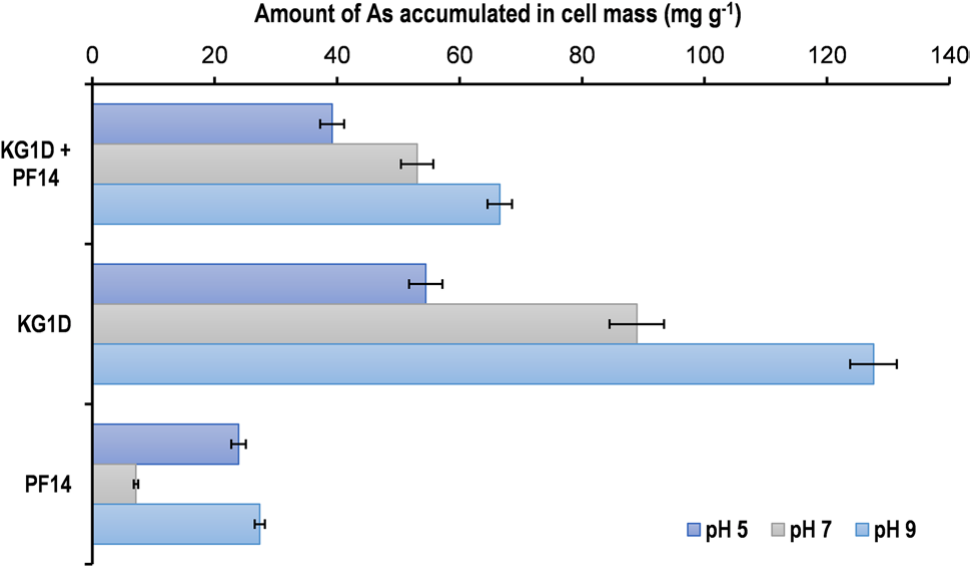
A comparative representation of As bioaccumulation by PF14 and KG1D, singly or cumulatively at different pH.

The amount of As bioaccumulated by both the bacterial isolates is depicted in Fig. 4, and it demonstrates that KG1D cell mass can efficiently accumulate relatively higher amounts of As at different pH. KG1D bioaccumulated 54.47 mg g^−1^, 88.95 mg g^−1^, and 127.60 mg g^−1^ of As at pH 5, 7 and 9, respectively, while PF14 bioaccumulated 23.90 mg g^−1^ of As at pH 5, 7.14 mg g^−1^ at pH 7 and 27.37 mg g^−1^ of As at pH 9. When both the isolates were used together, the amount of As bioaccumulated was 39.18 mg g^−1^ at pH 5, 53.06 mg g^−1^ at pH 7, and 66.58 mg g^−1^ at pH 9.

The metal removal and bioaccumulation efficiency of the isolates at different pH showed that KG1D is much more efficient in varying pH than PF14. KG1D accumulated and removed As efficiently at both acidic and alkaline pH. At neutral pH, PF14 was inefficient in removing metal from the culture medium but showed better efficiency only at acidic pH and moderate efficiency at alkaline pH. The efficiency of metal removal by PF14 changed with the change in media pH, i.e., it is primarily pH-dependent. This suggests that heavy metal removal from the surroundings differs from bacterial species to species^39^, and pH has a crucial role in removing As ions from bacterial cells^12^. When grown cumulatively, it was observed that the metal removal efficiency was not summative. This indicates that the pH of culture media affects the ionization state of metal ions and various functional groups on the bacterial surface by ionizing them and changing the overall bacterial surface charge. At acidic pH, the positively charged metal ions can easily interact with the bacterial surface as the metal ions are stable^40^, leading to the removal of As(III) by bacterial cell masses. But again, at alkaline pH, a further rise in metal removal by bacteria might be due to the deprotonation of metal binding sites in bacterial (biosorbents) cell walls originating due to the lower number of protons in the growth media^41^.

### As(III) biosorption potential of the bacterial biomass

Adsorption is an amalgamation of various methods, such as forming stable complexes, chelation, exchange of ions, redox reactions, physical adsorption, and surface precipitation^42^. Generally, biosorption is efficient over energy-consuming processes to remove heavy metals from the environment^43^. The adsorption of As(III) by KG1D and PF14 biomass was analyzed using the Langmuir isotherm model. The Langmuir adsorption isotherm model is used to evaluate the biosorption mechanism for metals in solutions^44^. Adsorption isotherms help to assess the biosorbents’ adsorption capacities and the nature of biosorption^45^. The maximum adsorption capacity or *q*_*max*_ of KG1D and PF14 biomass for As(III) was estimated to be 30.03 mg g^−1^ and 28.08 mg g^−1^, respectively. The R_L_ value for KG1D (0.41) and PF14 (0.22) was found to lie between 0 and 1, suggesting favourable monolayer adsorption of As ions on the cell surface. The adjusted regression coefficient (*R*^2^) for the Langmuir model was 0.95 for KG1D and 0.98 for PF14, suggesting a good fit. The relatively higher *q*_*max*_ of KG1D compared to PF14 indicates that KG1D may possess more adsorption sites on the cellular surface. Previous studies have shown that As(III) adsorption on cellular surfaces of biosorbents differs significantly. Studies using *Enterococcus faecalis*, *Escherichia coli*, and *Bacillus subtilis* untreated biomasses have shown no As(III) biosorption even after 72 h incubation^46^, while biomass of *Exiguobacterium profundum* PT2 has been found to adsorb As(III) at the rate of 25.2 mg g^−1^ of biomass^47^. About 31.09 mg As(III) per gram biomass biosorption has been reported for untreated biomass of a *Yersinia* sp. strain SOM-12D3^44^. In contrast, a *Bacillus cereus* strain SZ2 isolated from As-laden gold mine was found to adsorb about 153.41 mg g^−1^ of As(III)^45^. Though isolated from uncontaminated sites, the bacterial strains in our study were found to adsorb As(III) favourably.

### Metabolic profiling through FTIR

**Figure 5:**
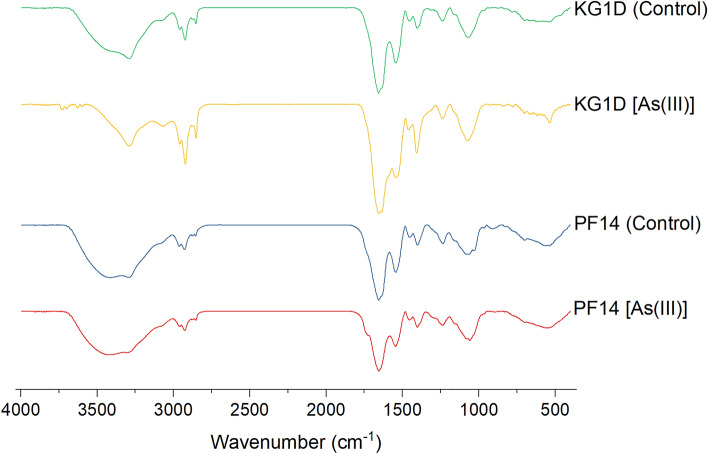
FTIR-based metabolic profiling of the isolate KG1D and PF14 in both control (metal-devoid) and As(III) supplemented growth medium at pH 9.

The spectra obtained through FTIR before and post-exposure to As(III) were compared for both isolates. It was found that both showed few specific changes (after heavy metal exposure) with respect to their control spectra (Fig. 5). The spectra for KG1D and PF14 showed a variable mixture of broad, sharp peaks or bands at different regions. For KG1D, the absorbance peaks were around 3289.10 cm^−1^, 2921.59 cm^−1^, 1655.41 cm^−1^, 1544.20 cm^−1^, 1460.06 cm^−1^, 1407.21 cm^−1^, 1236.64 and 1075.83 cm^−1^ post-exposure to As(III). These absorption peaks shifted from their control spectra that were in the regions of 3291.01 cm^−1^, 2923.67 cm^−1^, 1655.99 cm^−1^, 1543.96 cm^−1^, 1454.09 cm^−1^, 1402.32 cm^−1^, 1237.54 cm^−1^ and 1066.35 cm^−1^. The spectral bands for PF14 were shifted from 3413.32 cm^−1^, 2927.01 cm^−1^, 1655.69 cm^−1^, 1544.43 cm^−1^, 1401.28 cm^−1^, 1234.60 cm^−1^, and 1080.12 cm^−1^ to 3427.14 cm^−1^, 2925.63 cm^−1^, 1655.52 cm^−1^, 1545.44 cm^−1^, 1402.22 cm^−1^, 1236.77 cm^−1^, and 1058.49 cm^−1^ respectively, post exposure to As(III). The absorption bands around 3700–3000 cm^−1^ are due to O–H stretching, indicating the presence of OH groups. The 3000–2800 cm^−1^ spectral band exhibits vibrational stretching of C-H^48,49^, thus corresponding to CH_3_ and CH_2_ groups found in lipids. The intense peaks at 1655 cm^−1^ and 1544 cm^−1^ are due to the stretching of C=O of amides in cellular proteins and the latter due to N–H deformation of amides. The peak around 1453 and 1407 cm^−1^ indicates amide III groups and deprotonated carboxyl groups due to the bending vibration of C-N and asymmetric stretching of C–O. The peak found at 1236 cm^−1^ signifies P=O stretching existing in phosphoryl groups and phosphodiesters in nucleic acid. Lastly, the peak at 1075 cm^−1^ is connected with symmetrical stretching vibration of P=O found in nucleic acid and phosphorylated proteins^50,51^.

On comparing the FTIR spectrum of both strains after metal exposure, distinct differences were detected in the intensity of peaks and wavenumbers. Different peaks are visible mainly in four regions in the spectra of both isolates: one in the spectral range of 3000–2800 cm^−1^, second in 1750–1500 cm^−1^, and next in the range of 1500–1200 cm^−1^ and 1200–900 cm^−1^. These spectral regions highlight the different biomolecule classes in bacterial cellular surfaces^52^.

In the lipid region^53^ (3000–2800 cm^−1^), distinct variations are visible between the two bacterial strains: KG1D with three sharp peaks (2958.3, 2921.59, and 2851.76 cm^−1^) but three short absorption peaks for PF14 (2956.71, 2925.63 and 2848.70 cm^−1^). The absorption peaks for KG1D in the protein region^53^ (1750–1500 cm^−1^) appeared to be more or less the same as those observed for PF14. KG1D depicts two sharp and distinct maxima at 1655.41 cm^−1^ and 1544.20 cm^−1^, whereas the peaks (1655.52 and 1545.44 cm^−1^) observed for PF14 are less sharp than KG1D. Further, it can be seen that the absorption spectra in mixed or combined region^54^ (1500–1200 cm^−1^) of KG1D are characterized by a similar absorption band, but the wavenumbers that appeared were different compared to PF14. Three maxima (1460.06, 1407.20, and 1236.64 cm^−1^) can be seen in the case of KG1D; for PF14, the absorption bands were at 1448.46, 1402.22 and 1236.77 cm^−1^. Absorption peaks were also noticeable in the polysaccharide region^53^ (1200–900 cm^−1^); both the isolates have intense peaks, one at 1058.49 cm^−1^ for PF14, which in the case of KG1D appeared at 1075.83 cm^−1^.

On exposure to heavy metals, bacteria naturally adapt to the stressor by altering their molecular fingerprint and adopting various survival strategies. In some groups, there may be elevation in the synthesis and expressions of varied cellular proteins and enzymes that help to detoxify or prevent the entry of toxic heavy metals. Some even pump out the heavy metals that have gained access inside the bacterial cell, and some conceal those heavy metals in special sequester vesicles^52^. The spectral variations of both the isolates suggest differences in the metabolic fingerprints when grown in As contaminated environment. The metabolic profile of both isolates demands further probing using untargeted metabolomics, which could shed light on the presence of differentially expressed metabolites that might play a vital role in tolerating As.

### Whole genome sequence analysis and determination of As resistance genes

**Figure 6:**
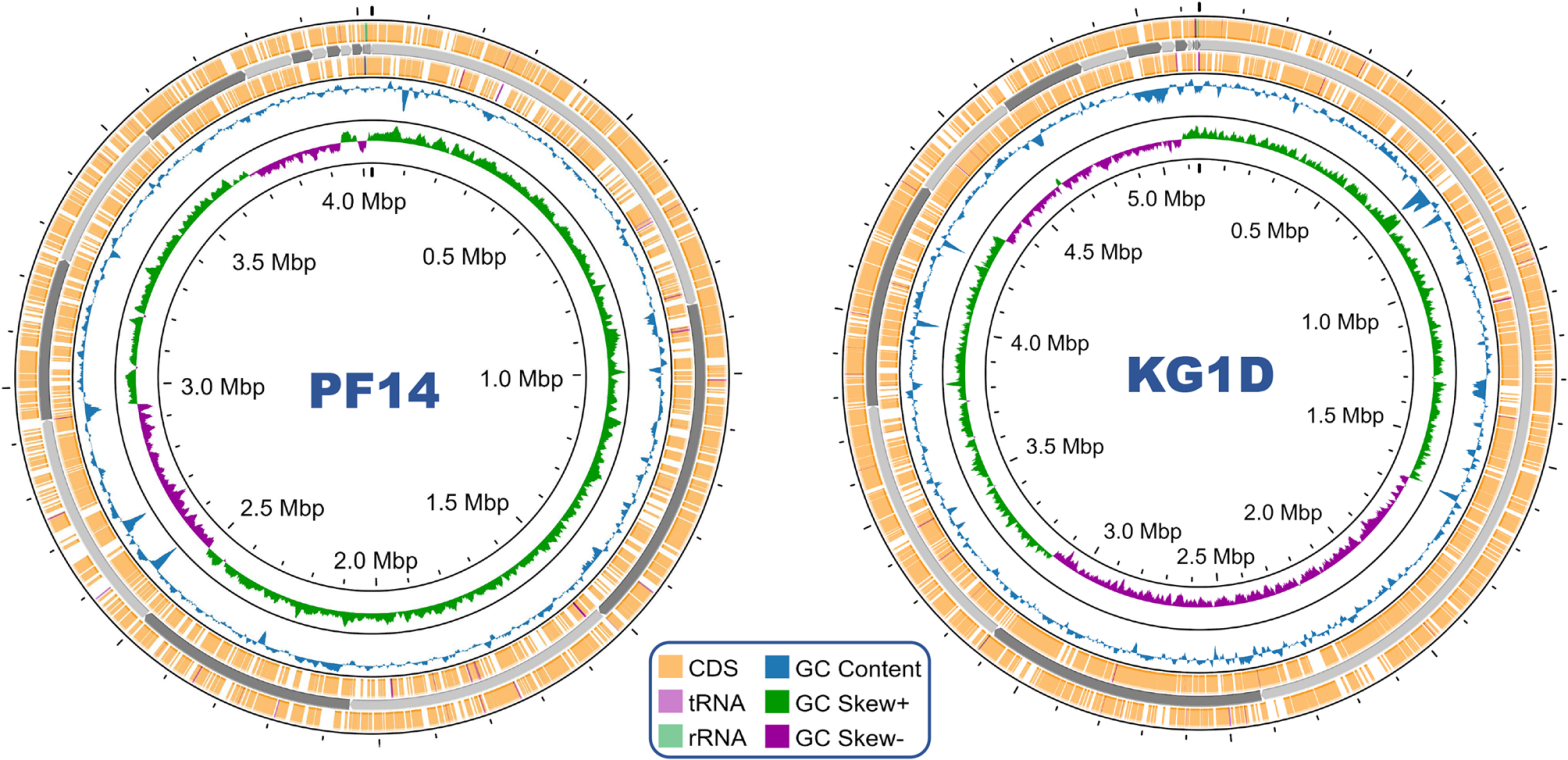
A circular representation of the genome maps of the arsenic resistant bacterial isolates PF14 (on the left) and KG1D (on the right) depicting the CDS, different RNA features, genome size, GC content and GC skew.

The whole genome sequence analysis of PF14 and KGID depicted a genome size of 4,025,851 and 5,141,233 bp, respectively. The GC content of PF14 was found to be 56.44%, while that of KG1D was 59.65%. A brief account of the different genomic features of both genomes is given in Table 3. The circular genome map of both the isolates is shown in Fig. 6. Genome annotation of PF14 and KG1D depicted 93.74% (3774 CDS) and 96.51% (4962 CDS) protein-encoding feature coverage, respectively.

**Table 3:** Brief details regarding genome sequencing, assembly and structural annotation of the PF14 and KG1D whole genomes.

Features	Whole Genome Sequence	
	PF14	KG1D
Number of raw reads	14,131,632	9,609,050
Reads after QC	13,372,002	8,310,200
Read length	151	151
Contigs N50 (bp)	535,900	704,804
Contigs L50	2	3
Genome size (bp)	4,025,851	5,141,233
GC%	56.44	59.65
CDS	3774	4962
Protein-Encoding Feature Coverage	93.74%	96.51%
tRNA	53	78
rRNA	03	03

To tolerate As, many bacteria possess a polycistronic arsenic operon (*ars*) comprising of *arsR*, *arsB* and *arsC* genes, which endow the bacteria with arsenic resistance by encoding ArsR, ArsB and ArsC proteins, respectively. The presence of these three genes has been established as minimal and sufficient criteria for arsenic tolerance^55^. The *arsR* gene is a trans-acting transcriptional regulator of the arsenic resistance operon, which encodes a repressor protein. It controls other genes of the *ars* operon as well as its own expression. Bacterial transcription regulatory proteins bind to DNA via a helix turn helix motif (HTH) with a well-characterized number of conserved residues residing in the central part of the protein. The ArsR regulatory protein also possesses residues for binding metal- oxyanions such as arsenite, antimonite and arsenate. The ArsR protein has been reported to occur in multiple copies, suggesting the occurrence of multiple arsenic operons in an organism^56^. The *arsB* gene encodes for ArsB protein, the arsenical pump membrane protein or the arsenic transporter. Extrusion of the toxic arsenite from the cell in simpler *arsRBC* operons is driven by the membrane potential or the proton motive force primarily by the ArsB protein^57^. The *arsC* gene encodes a glutaredoxin-dependent arsenate reductase, which transforms arsenate into its extrudable form arsenite. In moderately reducing and oxidizing atmospheric conditions, the prevalent arsenic form varies. In the former condition, arsenite is observed, while arsenate species dominate in oxygen-rich environments^58^.

Whole genome sequence analysis of the strains PF14 and KG1D confirmed the presence of an entire array of genes related to arsenic tolerance. Genome mining revealed the presence of the *arsRBC* genes in both PF14 and KG1D. The obtained nucleotide and protein sequences were subjected to a two level in silico validation for corroborating the presence of *arsRBC* genes encoding the ArsR, ArsB and ArsC proteins. The gene length and genomic position of the *arsRBC* genes in PF14 and KG1D genomes have been provided in Table 4. The obtained nucleotide sequences of the *ars* genes were subjected to BLASTX (Translated protein blast), which would translate in all six reading frames to validate the genes encoding for the presumed protein sequence. The compiled BLASTX results of the top two hits for the genes *arsB*, *arsC* and *arsR* are provided in the supplementary Table S4. The *arsB* gene of PF14 and KG1D showed maximum sequence identity with *Alcaligenes* (99.75%) and *Serratia* (100%), respectively. Similarly, the *arsC* gene of PF14 and KG1D showed significant identity with that of *Alcaligenes* (100%) and *Serratia* (100%), respectively. Similar identities (100%) were obtained for PF14 and KG1D with *Alcaligenes* and *Serratia*, respectively, using the different *arsR* coding sequences. Finally, InterProScan was used to validate further the protein sequences encoded by the *arsRBC* genes of PF14 and KG1D and determine their family, homologous super family, domains, molecular function, biological process and important sites. The results show that the *arsB* gene of both the isolates encodes the arsenic transporter protein belonging to the Arsenical pump ArsB family containing the ArsB permease domain. Similarly, the *arsC* gene encoded the arsenate reductase (glutaredoxin) protein containing the ArsC domain and is grouped under the arsenate reductase-like family and thioredoxin-like superfamily. All four copies of the *arsR* gene of both the isolates PF14 and KG1D were found to encode the metalloregulator ArsR/SmtB family transcription factor containing the helix turn helix ArsR DNA binding domain (HTH_ArsR_DNA_bd_dom) belonging to the winged helix-like DNA binding domain (Winged Helix- like _DNA-bd_dom) superfamily. The presence of the genomic *arsR*, *arsB* and *arsC* coding sequences in the chromosomal DNA of the aerobic bacteria PF14 and KG1D thus corroborates their genotypic potential to tolerate and resist different inorganic forms of arsenic.

**Table 4:** The gene length along with start and stop positions of all the *arsRBC* genes detected in the genome of the As resistant isolates PF14 and KG1D.

Isolate	Gene name	Gene length	Genomic location	
			Start position	Stop position
PF14	*arsB*	1290	212,496	211,207
	*arsC*	432	211,184	210,753
	*arsR1*	324	212,908	212,585
	*arsR2*	327	506,375	506,686
	*arsR3*	354	55,708	56,034
	*arsR4*	312	861,605	861,252
KG1D	*arsB*	1290	2,304,900	2,303,611
	*arsC*	432	2,303,596	2,303,165
	*arsR1*	327	2,305,296	2,304,970
	*arsR2*	684	1,123,528	1,123,842
	*arsR3*	318	1,039,034	1,039,351
	*arsR4*	315	671,609	670,926

## Materials and methods

### Isolation and screening of indigenous As-tolerant bacteria

As-tolerant bacteria were isolated from the Kulik River and Kulik forest, located along the stretch of the Kulik River on the outskirts of Raiganj town in West Bengal, India. The details of the sampling sites are given in supplementary Table S2. To isolate bacteria from the collected soil and water samples, serial dilution followed by pour plating was carried out. In this technique, soil sample suspension was made by adding 1 gm of soil with 10 mL of double distilled sterilized water (stock solution) and then vigorously shaking for approximately 1 min. The solution was then left to sediment for a short period. The stock solution was diluted serially from 10^−1^ to 10^−4^. From each tube, 0.1 mL of the diluted solution was pour plated into nutrient agar (NA) medium^59^ supplemented with 100 µg mL^−1^ of As(V) in sodium arsenate form^60^ and was incubated (OVFU, OBIS-1518D) under aerobic conditions at 35 °C for 24 h and the plates were observed at regular interval for the presence of bacterial growth on the culture media. For the river water, samples were serially diluted from 10^−1^ to 10^−4^ order using sterile double distilled water^61^ and were then plated as mentioned above. When bacterial colonies were found to grow successfully, a single solitary colony was sub-cultured repeatedly by streaking in NA slants followed by incubation at 35 °C for 24 h to achieve profuse growth. Bacteria were isolated based on the colony morphology on a solid medium. Then, the As-tolerant isolates were preserved in a 1:1 solution of 30% glycerol and nutrient broth (NB) at -20 °C for future use.

### Determination of pH, temperature, and As content of the samples

The pH and temperature of the collected samples were determined on-site using a portable digital pH and temperature meter (Hanna, model HI991003P). For the detection of As content in soil, 0.5 gm of soil sample was taken in a Teflon beaker, and 10 mL hydrofluoric and 5 mL nitric acid (65% v/v) was added to it. The solution was dried by heating on a hot plate, mixed with 10 ml of aqua-regia and again heated to dissolve the dry mass in solution. The obtained solution was diluted with 100 ml deionized H_2_O^62^ and run through a Simultaneous ICP Spectrometer (SPECTRO Analytical Instruments GmbH, Germany, ARCOS). To estimate arsenic concentration in water samples, 15 mL of each sample was acidified with ultrapure nitric acid (65% v/v) until the pH of the solution became less than 2. The solution obtained was filtered using syringe filters with a pore size of about 0.45 µm and run through the ICP Mass Spectrometer (Thermo Fisher Scientific, Element XR).

### Determination of MIC for As

Each isolated strain was first allowed to grow in 10 mL of minimal salts medium (MSM) in an orbital shaking incubator (SVIS-301 M) at 120 rpm and 35 °C. After 24 h of growth, an inoculum of 0.1 mL of culture from each strain was then transferred to sterile Erlenmeyer flasks containing 20 mL MSM supplemented with graded concentrations of As in the form of sodium arsenite (NaAsO_2_) and sodium arsenate (Na_2_HAsO_4_.7H_2_O) ranging from 100 to 2000 µg mL^−1^. The MSM used in our study, comprised of Na_2_HPO_4_ 4.5 g L^−1^, KH_2_PO_4_ 1.5 g L^−1^, NH_4_Cl 0.3 g L^−1^, MgSO_4_ 0.1 g L^−1^, yeast extract 0.5 g L^−1^, and C_6_H_12_O_6_ 5.0 g L^−1^. The pH of MSM used for this experiment was adjusted around 7.2 ± 0.2 at 25 °C. Two control setups were also kept aside. The negative control consisted of an Erlenmeyer flask containing MSM supplemented with As(III) and As(V) solution without bacteria. The positive control consisted of MSM and bacteria without As(III) and As(V). The above experimental setup was kept in an incubator at 35 °C, shaking at 120 rpm for 72 h. The entire experiment was carried out with three replicates. The MIC was studied by measuring O. D. at 600 nm after 72 h using a UV–visible spectrophotometer (Analytical, UV-2080 T).

### Determination of MBC to As

Experiments were conducted by culturing isolates in MSM media mixed with As(III). The experimental setup with their respective negative controls was kept in a shaking incubator at 35 °C. The O. D. was measured at every 24 h interval to check viability. If bacteria were found to show inconspicuous growth, aliquots of 0.1 mL were transferred from this MSM supplemented with As(III) to fresh MSM medium lacking the heavy metal, and bacterial growth was checked by recording O. D. (600 nm) at 24 h intervals up to 48 h. The absence of visible growth in fresh MSM media even after the set time frame depicts that the concentration is the MBC value for the element (As). On the other hand, if the MSM medium turned turbid due to the growth of the bacteria, it indicates that the concentration or dose of heavy metal needs to be elevated in the growth medium to achieve a concentration where the bacteria would fail to show any sign of visible growth.

### Determination of MIC against other heavy metals

MIC determination for other heavy metals was done following the protocol used for As. For determination of tolerance to other heavy metals, graded concentrations of salts of copper (CuSO_4_·5H_2_O), cadmium (CdCl_2_), nickel (NiSO_4_·6H_2_O), zinc (ZnSO_4_·7H_2_O), mercury (HgCl_2_) and chromium (K_2_Cr_2_O_7_) were supplemented in growth media. All the metal salts and their formula used during this study are listed in the supplementary Table S5.

### Morphological and biochemical characterization of As-tolerant bacteria

The morphology of the tolerant isolates was studied using Gram staining and SEM. Fresh cultures of 18 h were used for Gram staining using the Gram staining kit (Himedia, India). A compound microscope with a bright field (Olympus, CH20i) was set up to examine the cellular structure of the chosen isolates^10^. Finally, SEM was performed for a more detailed study of cellular structure both in control and in presence of As(III) supplemented Luria Broth (LB) medium (at 300 µg mL^−1^). For SEM, fresh overnight-grown bacterial broths were centrifuged (REMI C-24 Plus) at 10,000 rpm for 10 min to achieve sufficient biomass. Then, after washing with phosphate buffer saline (PBS; 7.2 ± 0.2 pH) 2–3 times, the pellets were fixed with 2.5% glutaraldehyde solution. Finally, the cell mass was passed through increasing concentration of alcohol (10–100%) to remove any trace of water. After dehydration, the samples were coated with gold particles and observed under an Environmental Scanning Electron Microscope (Carl Zeiss, EVO 18, version 6.02).

### Molecular identification of the As-tolerant isolates using 16S rRNA gene sequencing

Molecular identification of the As-tolerant isolates was performed by 16S rRNA gene sequencing. First, total DNA was extracted following the phenol–chloroform method. Then, the purified 16S rRNA genes were amplified^63^ through the use of universal primers 16F27 [5′–CCA GAG TTT GAT CMT GGC TCA G–3′] and 16R1492 [5′–TAC GGY TAC CTT GTT ACG ACT T–3′]. The amplified 16S rRNA gene PCR product was purified by PEG-NaCl precipitation, and the product was directly sequenced on an ABI 3730XL automated DNA sequencer. BioEdit 7.2 (available at https://bioedit.software.informer.com/7.2/) was used to perform the sequence assembly. Post-acquisition of sequences, the 16S rRNA gene sequences were deposited in the GenBank repository to obtain the accession numbers.

To identify the isolates, a nucleotide BLAST^64^ of the acquired sequence against the 16S ribosomal RNA sequences database containing curated type strain sequences from bacteria was performed^65^. The type strain demonstrating maximum sequence identity with the obtained 16S rRNA gene sequences was further validated using the List of Prokaryotic names with Standing in Nomenclature^66^ (LPSN) available at https://lpsn.dsmz.de (accessed on 11th July 2023).

### Study of growth kinetics in the control and As supplemented media

The growth kinetics of the potent isolates, both in the presence and absence of As(III) and As(V), were determined in batch culture. 0.1 mL of bacterial inoculum from a fresh culture was transferred to 100 mL Erlenmeyer flasks containing 20 mL of freshly prepared MSM supplemented with As(III) and As(V) at concentrations equivalent to the MTC of both the isolates. A positive control setup containing only the isolates in MSM and a negative control (MSM supplemented with As(III) and As(V) were also kept to compare the growth with the As-stressed condition. All the experiment was carried out in triplicates, and the Erlenmeyer flasks were kept in a shaking incubator at 35 °C at 120 rpm. The bacterial growth was measured by recording O. D. at 600 nm using a spectrophotometer (Analytical, UV-2080T) every two hours until the cultures reached the stationary growth phase. Lastly, the growth curves were prepared by plotting the O. D. values on the ordinate and time on the abscissa, and the growth rate and generation time of the isolates were calculated.

### The cumulative effect of pH and metal stress on the growth of bacteria

To study the effect of varying pH on bacterial growth, the growth of the isolates was monitored in MSM with pH ranging from 4 to 11. Bacterial cultures were grown in 100 mL Erlenmeyer flasks containing 20 mL of sterile MSM. In the medium, 0.1 N HCl or 1.0 M NaOH solution was added as pH regulators to get the desired pH and then finally measured with a pH meter (Labtronics, CE LT-50). The isolated bacterial strains were pipetted onto the medium with varying pH. Each experimental setup was arranged in triplicates with and without As supplements. The As concentration was set as per the result obtained from the MIC studies, and then all the flasks were maintained for about 48–72 h at 35 °C, shaken at 120 rpm using an orbital shaking incubator. Bacterial growth was measured by recording O. D. at 600 nm using a spectrophotometer (Analytical, UV-2080T).

### Screening for arsenite oxidizing ability

The bacteria were pre-cultured overnight at 35 °C in a chemically defined medium (CDM)^67^. After that, bacterial suspension was spread uniformly on CDM agar-containing culture plates (the experiment was repeated thrice, each time with ten replicates for each isolate). The CDM used comprised of K_2_HPO_4_ 10.0 g L^−1^, Na_2_SO_4_ 1.0 g L^−1^, NH_4_Cl 1.0 g L^−1^, MgSO_4_ 2.0 g L^−1^, CaCl_2_·2H_2_O 6.5 g L^−1^, Fe_2_SO_4_·7H_2_O 0.4 g L^−1^, and NaHCO_3_ 0.8 g L^−1^. The pH of CDM used for this experiment was adjusted around 7.2 ± 0.2 at 25 °C. After 18–20 h of visible growth at 35 °C, 0.1 M AgNO_3_ solution was poured on the agar plates to submerge the colonies^68,69^. The reaction between AgNO_3_ and arsenite or arsenate leads to the formation of a coloured precipitate. The appearance of a brown precipitate indicates the existence of silver arsenate (Ag_3_AsO_4_) in the agar plate. On the contrary, a yellowish-white deposit suggests the presence of silver arsenite (Ag_3_AsO_3_)^68^.

### Estimation of As removal and bioaccumulation ability

Erlenmeyer flasks containing 50 mL of MSM supplemented with 300 µg mL^−1^ of As(III) and with pH 5, 7 and 9 were used to carry out this experiment. About 0.1 mL of an aliquot from freshly grown overnight culture was poured in sterile MSM supplemented with As (300 µg mL^−1^) at different pH and was kept at 35 °C (the optimal growth temperature of the isolates), at 120 rpm, in an orbital shaking incubator until growth reached the mid-log phase. To check if there is any additive effect on As uptake by the selected isolates, both the isolates were inoculated concomitantly in As-supplemented MSM with different pH (pH 5, 7, and 9) at 35 °C, 120 rpm in an orbital shaking incubator. In all cases, negative or abiotic controls (MSM media and As(III)) lacking bacterial inoculum were also set to observe whether metal uptake is purely by live bacterial cells and not by metal precipitation due to the interaction between medium constituents and metal ions [As(III)]^70^. Next, the culture containing bacterial cell masses was centrifuged (REMI-C-24 Plus) at 12,000 rpm at 4 °C for 10 min. Supernatants were kept aside in a clean and sterile flask for acid digestion. The obtained cell pellets were washed with 50 mL of 0.1 M HCl to remove metal ions adsorbed on live cell masses. Centrifugation was done at 12,000 rpm at 4 °C for 10 min. The acidified supernatants were collected and stored in flasks for acid digestion. Finally, the pellets were dehydrated in a hot air oven at 40–45 °C for 12–18 h; their final dried mass was measured and was digested with ultrapure nitric acid (65% v/v)^71^. The concentration of As removed from the culture media and bioaccumulated within intracellular compartment^31^ was measured using a Simultaneous ICP Spectrometer (SPECTRO Analytical Instruments GmbH, Germany, ARCOS). The amount of metal bioaccumulated^31^ by these strains from the tested metal solution was determined by the equation,

$$Bioaccumulation \left( {{\text{mg g}}^{ - 1} } \right) = \left[ {V\left( {C_{i} - C_{f} } \right)} \right]/M$$

where *C*_*i*_ denotes the concentration of the metal ions in solution initially (mg L^−1^), *C*_*f*_ represents the final concentration of metal ions left in solution (mg L^−1^), *V* represents the volume of the solution (L), and *M* is the dry weight of the biomass (g). The obtained data was analyzed statistically by one-way analysis of variance (1-ANOVA) using SigmaPlot (version 12.0) to find out if there exists any significant difference in As removal by the different isolates either singly or cumulatively.

### Biosorption capacity of the isolates and adsorption isotherm evaluation

Biosorption experiments were performed, and adsorption isotherm was evaluated to assess the biosorption capacity of the isolates. The culture solution was prepared by inoculating the bacteria in 1000 mL LB medium and incubating at 35 °C, with a shaking rate of 120 rpm. The culture was grown until it reached the late exponential phase. The overnight grown culture was centrifuged (REMI-C-24 Plus) at 12,000 rpm at 4 °C for 10 min. The pellets obtained were washed with 1X PBS (7.2 ± 0.2 pH) three to four times and were finally dehydrated in a hot air oven at 40–45 °C for 12–18 h. For each run, 50 mL sterile deionized water amended with graded concentrations of As(III) was added in 250 mL Erlenmeyer flasks. The concentration of As(III) used in this study ranged from 25 to 125 µg mL^−1^. Then, a fixed dose of about 1 mg mL^−1^ of the dried cellular mass was added to As enriched sterile water. The solution pH was kept at 7, since maximum As(III) biosorption is known to occur at neutral pH^44,72,73^. Each flask was incubated at 35 °C till 24 h, and the pellets were collected by centrifugation. Residual As ions were then determined in the supernatant solution using a Simultaneous ICP Spectrometer (SPECTRO Analytical Instruments GmbH, Germany, ARCOS). Each treatment was run in triplicates, and negative control was also set to delineate metal precipitation from cellular biosorption^74^. The biosorption capacity at equilibrium for each bacterium was quantified according to the equation,

$$q_{e} = \frac{{C_{i} - C_{e} }}{W} \times V$$

where *q*_*e*_ denotes the amount of metal ions adsorbed per unit of biosorbent at equilibrium in mg g^−1^, *V* is the working volume in L, *C*_*i*_ is the initial concentration of metal ions (before adsorption) in mg L^−1^, *C*_*e*_ represents the equilibrium concentration of metal ions (after adsorption) in mg L^−1^, and *W* is the adsorbent weight in g^75^.

To better understand the metal biosorption process by the biosorbents, the data was evaluated using the Langmuir adsorption isotherm model^76^. This model states that effective monolayer adsorption occurs at particular homogeneous sites within the adsorbent. The Langmuir isotherm is expressed by the equation,

$$q_{e} = \left( {q_{max} K_{L} C_{e} } \right)/\left( {1 + K_{L} C_{e} } \right)$$

where *q*_*e*_ is the adsorbed amount of the metal ions (mg g^−1^), *q*_*max*_ is the monolayer adsorption capacity (mg g^−1^), and *K*_*L*_ represents the Langmuir adsorption constant (L mg^−1^). The separation factor or R_L_ value determines the nature and feasibility of the adsorption process^74^ and is expressed as $R_{L} = 1/\left( {1 + K_{L} C_{i} } \right)$. A favourable adsorption process has an R_L_ value between 0 and 1.

### FTIR-based comparative metabolic profiling of As-resistant cells

FTIR analysis was done to interpret changes in the expression pattern of functional groups and their respective macromolecules on the cell surface before exposure to As(III) and post-metal exposure. The functional groups and respective biomolecules of As(III) loaded and unloaded cells were analyzed using a Fourier-transform infrared (FTIR) spectrometer (make Bruker, Germany; model 3000 Hyperion Microscope with Vertex 80 FTIR System). The KBr disk method, in which sample disks are prepared from bacterial biomass blended with potassium bromide (KBr), was employed^36^. To gather abundant biomass, first, fresh overnight-grown bacterial broths were centrifuged (REMI C-24 Plus) at 10,000 rpm for 10 min. Then, after washing with 0.5% NaCl thrice, the pellets were dried in a hot air oven for 5–10 h at 45 °C to remove any traces of water. Finally, the dehydrated cell masses were ground to a fine powder in a mortar pestle, and 1 mg of it was mixed with 0.2 mg of KBr to cast a die pellet of 13 mm size. The pellet samples were scanned 64 times within the 4000–400 cm^−1^ range with a resolution of 4 cm^−1^. The spectra generated from the samples were recorded, processed using OPUS 8.2, baseline corrected, normalized, and analyzed to gain information about the changes in functional groups residing in various compounds^52^.

### DNA extraction, whole genome sequencing and assembly

Extraction of genomic DNA was performed using the QIAGEN DNeasy Blood and Tissue Kit (Cat. No. 69504) as per the protocol supplied by the manufacturer from the As tolerant bacterial strains freshly grown overnight in LB medium. Paired-end DNA library was prepared using the NEBNext Ultra II FS DNA Library Prep Kit for Illumina (Cat. No. E6177L) following the manufacturer’s instructions. DNA quantification was done using the Qubit DNA HS Assay Kit and library quality was assessed using Tapestation (Agilent). Whole genome sequencing was done on the Illumina NovaSeq 6000 platform^77^, at LifeCell International (P) Ltd., Bengaluru, India. Data pre-processing and quality control were also taken into consideration prior to assembly. The pre-trimmed data quality was checked using the FastQC^78^ and MultiQC^79^ tools. The data was checked for base call quality distribution, % bases above Q20, Q30, %GC, and sequencing adapter contamination. The raw sequence reads were processed to remove the adapter sequences and low-quality bases using the Fastp tool (v0.12.4)^80^. The post-trimmed QC passed reads were assembled de novo with the Unicycler tool (v0.5.0)^81^. During assembly, contigs shorter than 200 bp were removed from the assembly. The quality of the assemblies, such as N50, and the number of contigs were assessed with QUAST (v5.0.2)^82^. The processed reads were mapped back onto the assembled contigs to validate the assembled genomes using Bowtie 2 (v2.4.5)^83^. The genome completeness was checked using BUSCO (v5.3.2)^84^. The Whole Genome Shotgun projects containing the SRA data and assembled genome of the As-resistant isolates KG1D and PF14 have been deposited at DDBJ/ENA/GenBank under the accession JBAGIH000000000 (BioProject PRJNA1075445) and JBAGRS000000000 (BioProject PRJNA1075448), respectively.

### Genome annotation and detection of arsenic resistance genes

Genome annotation was performed using the RAST tool kit (RASTtk) from the Bacterial and Viral Bioinformatics Resource Center (BV-BRC) 3.34.11 web resource^85^ (https://www.bv-brc.org/). Circular genome maps were prepared using the online tool Proksee^86^ (https://proksee.ca/). The determinants linked to arsenic resistance were mined from the whole genome sequences after annotation, and the obtained gene sequences were subjected to validation and functional analysis using translated BLAST (BLASTX) and InterProScan (https://www.ebi.ac.uk/interpro/).

### Supplementary Information

Supplementary Figure S1.

Supplementary Figure S2.

Supplementary Figure S3.

Supplementary Figure S4.

Supplementary Table S1.

Supplementary Table S2.

Supplementary Table S3.

Supplementary Table S4.

Supplementary Table S5.

Supplementary Legends.

## Data Availability

The manuscript and the supplementary files provide all the data associated with this study.
