# Supplementary figures and images for: Isolation, characterization, identification, genomics and analyses of bioaccumulation and biosorption potential of two arsenic-resistant bacteria obtained from natural environments
Source: Sci Rep. 2024 Mar 8;14:5716. doi: 10.1038/s41598-024-56082-6 (PMC10924095; doi:10.1038/s41598-024-56082-6)

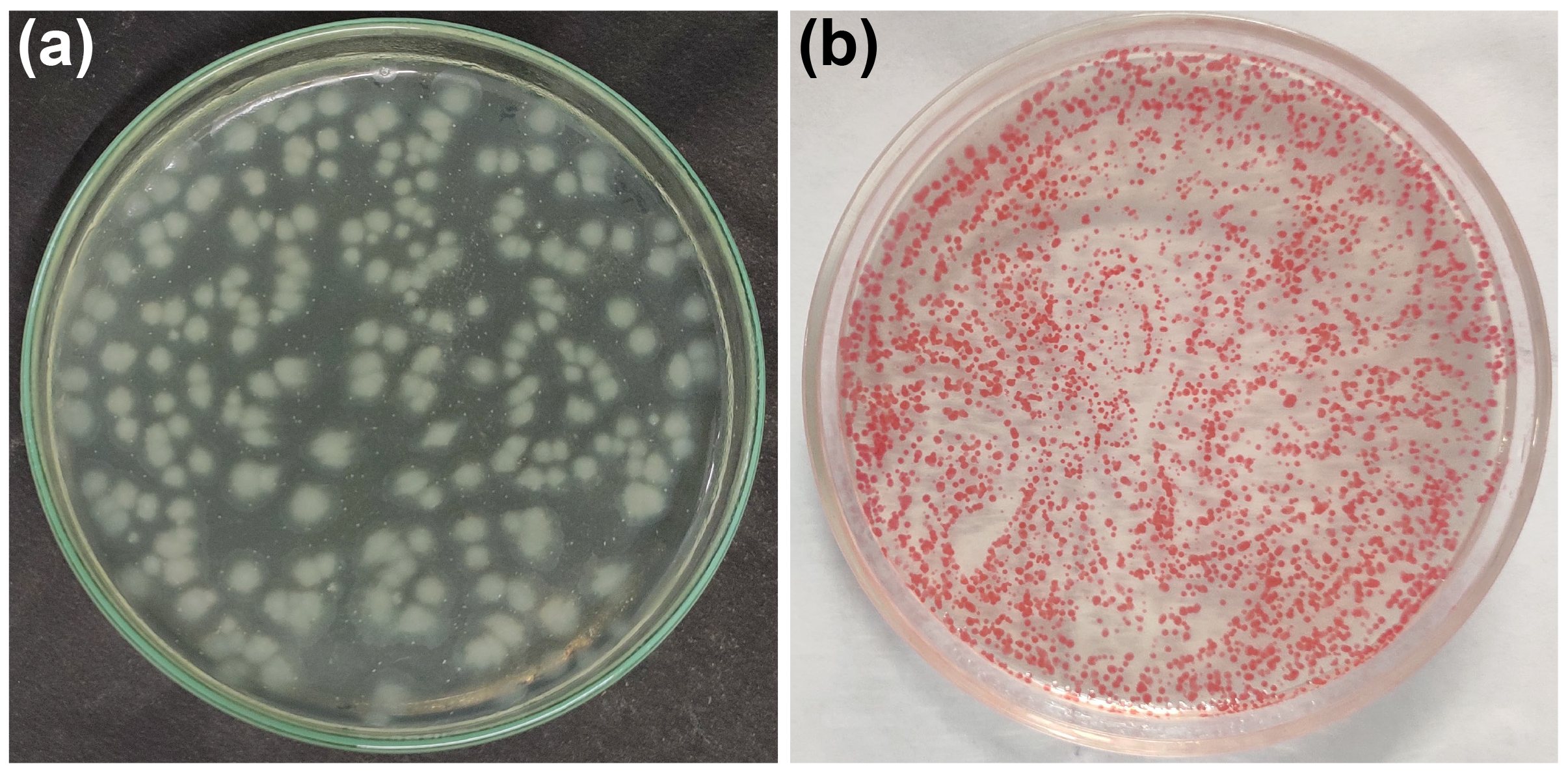

Supplement: Supplementary file 1 — Supplementary Figure S1. [file 41598_2024_56082_MOESM1_ESM.tif]

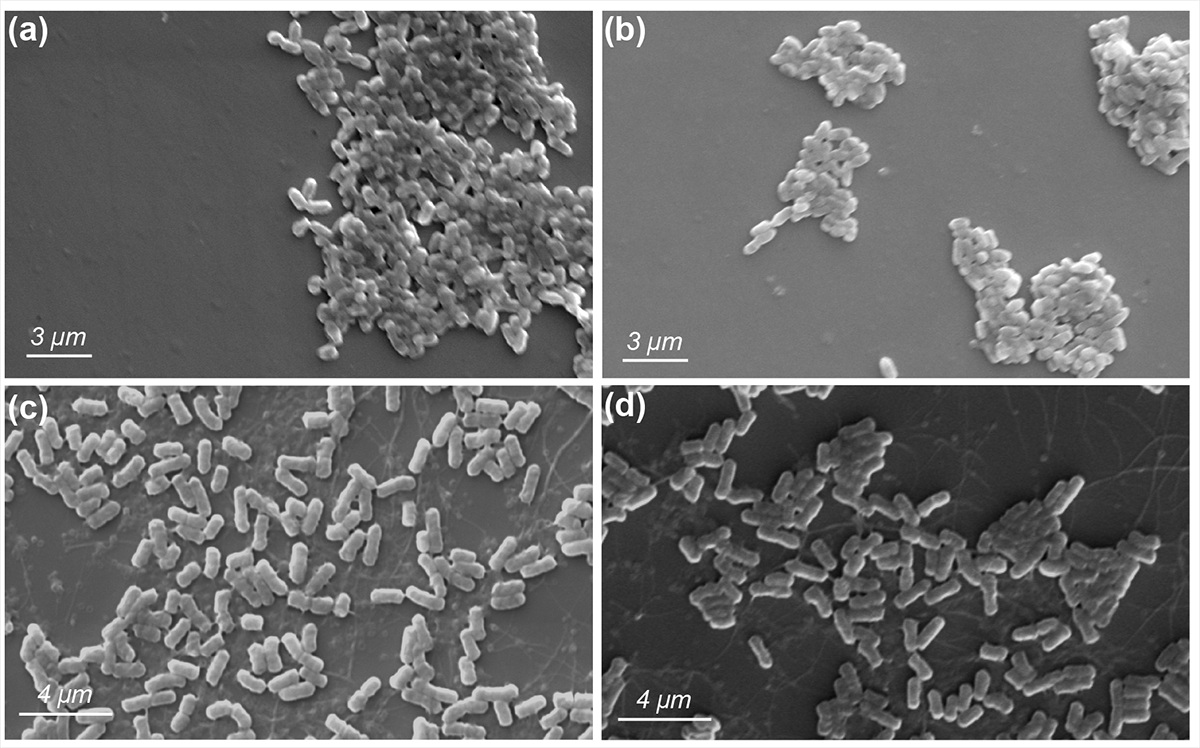

Supplement: Supplementary file 2 — Supplementary Figure S2. [file 41598_2024_56082_MOESM2_ESM.tif]

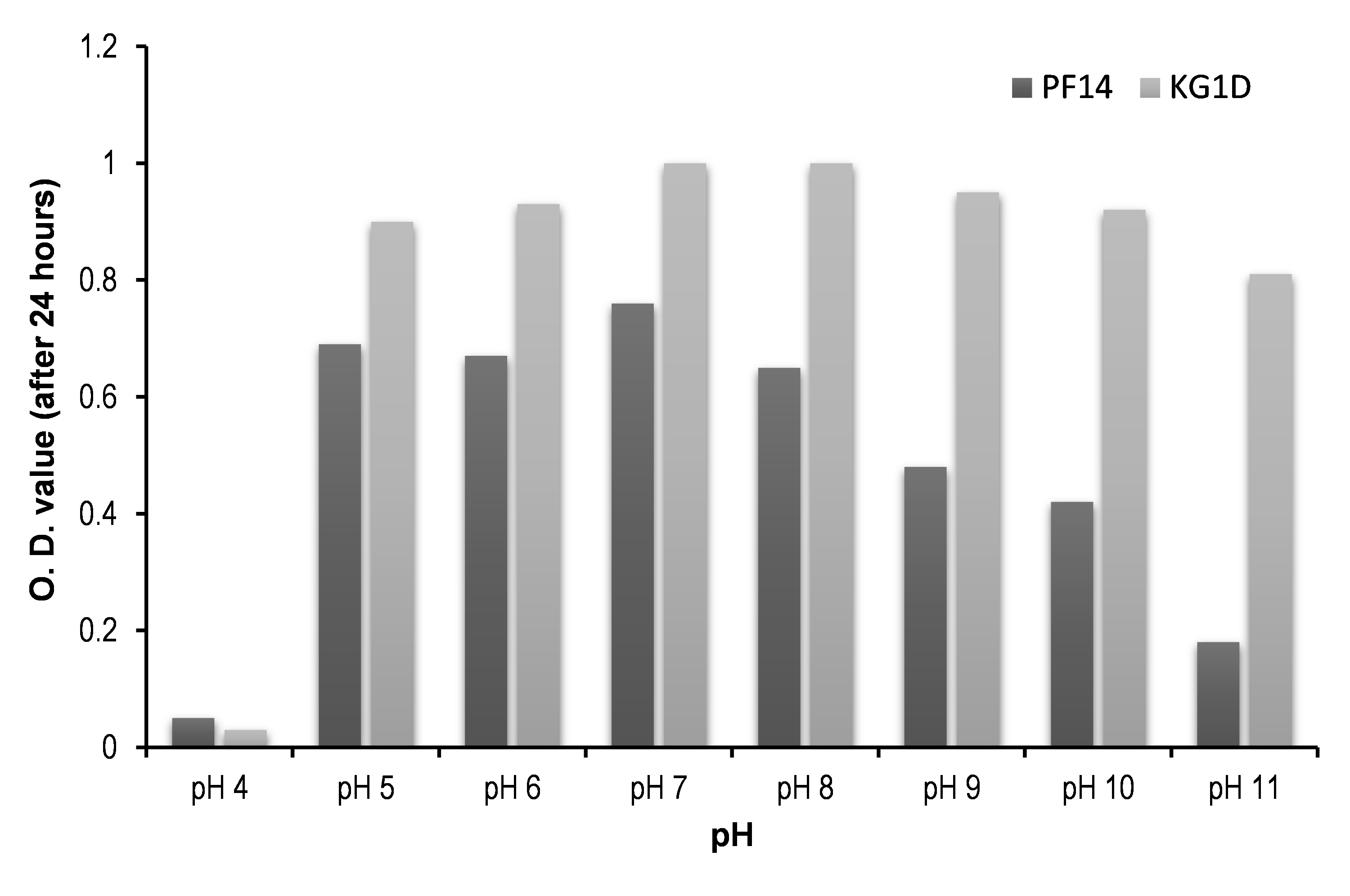

Supplement: Supplementary file 3 — Supplementary Figure S3. [file 41598_2024_56082_MOESM3_ESM.tif]

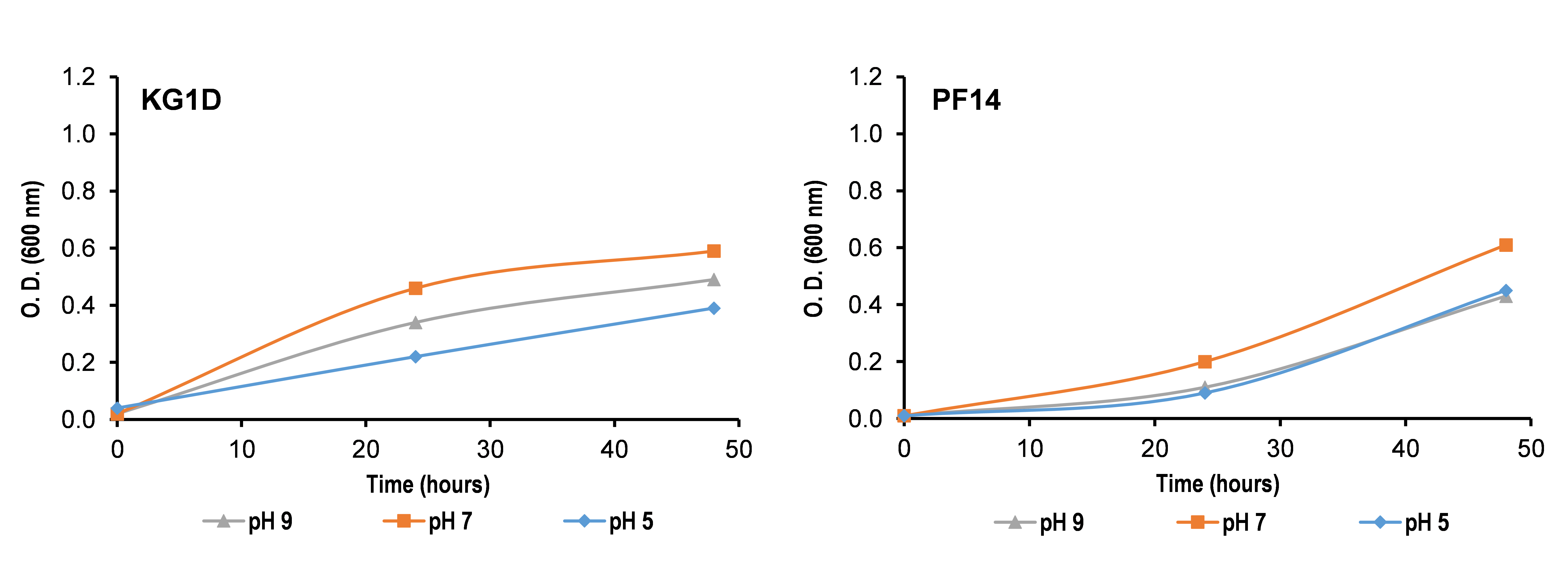

Supplement: Supplementary file 4 — Supplementary Figure S4. [file 41598_2024_56082_MOESM4_ESM.tif]
