# Supplementary material for: Isolation, characterization, identification, genomics and analyses of bioaccumulation and biosorption potential of two arsenic-resistant bacteria obtained from natural environments
Source: Sci Rep. 2024 Mar 8;14:5716. doi: 10.1038/s41598-024-56082-6 (PMC10924095; doi:10.1038/s41598-024-56082-6)
Supplement: Supplementary file 5 — Supplementary Table S1. [file 41598_2024_56082_MOESM5_ESM.docx]

**Supplementary Table S1** The details of isolates along with their MIC values of As(III) and As(V) obtained during this study.

| **Sl. No.** | **Name of Isolate** | **As(III)** | **As(V)** |
| --- | --- | --- | --- |
|  |  | **(µg mL^-1^)** | |
| 1 | K7 | 200 | 1,000 |
| 2 | KG1D | 600 | 1,800 |
| 3 | PF14 | 500 | 2,500 |
| 4 | K9 | 100 | 1,000 |
| 5 | K10 | 100 | 1,000 |
| 6 | K11 | 100 | 1,000 |
| 7 | K12 | 200 | 1,000 |
| 8 | I3 | 100 | 1,600 |
| 9 | I4 | 100 | 800 |
| 10 | I5 | 100 | 800 |
| 11 | KSS-1 | 100 | 1,000 |
| 12 | KSS-2 | 100 | 1,000 |
| 13 | KSS-3 | 100 | 1,000 |
| 14 | KSS-4 | 100 | 1,000 |
| 15 | KSS-5 | 100 | 1,000 |
| 16 | KSS-6 | 100 | 1,000 |
| 17 | KSS-7 | 100 | 1,000 |
| 18 | KSS-8 | 100 | 1,000 |
