# Supplementary material for: Isolation, characterization, identification, genomics and analyses of bioaccumulation and biosorption potential of two arsenic-resistant bacteria obtained from natural environments
Source: Sci Rep. 2024 Mar 8;14:5716. doi: 10.1038/s41598-024-56082-6 (PMC10924095; doi:10.1038/s41598-024-56082-6)
Supplement: Supplementary file 6 — Supplementary Table S2. [file 41598_2024_56082_MOESM6_ESM.docx]

**Supplementary Table S2** Details of the sampling sites including geographical location, pH, temperature and As content of the samples.

| **Sl. No.** | **Sampling sites id** | **Name of the locality** | **Geographical Coordinates** | **Source** | **pH** | **Temperature (°C)** | **As concentration** |
| --- | --- | --- | --- | --- | --- | --- | --- |
|  | K | Subhasganj | 25° 37´ 11´´N  88° 06´ 46´´E | Water | 7.20 | 31 | Not detected by ICP-MS  (or less than 0.01 ppm) |
|  | KG | Under Kulik Bridge (on NH-12) | 25° 38´ 06.7´´N  88° 07´ 19.9´´E | Water | 7.31 | 32 |  |
|  | I | Bhattadighi | 25° 39´ 09´´N  88° 08´ 37´´E | Water | 7.57 | 31 |  |
|  | PF | Kulik Forest | 25° 38' 20.39" N  88° 7' 12" E | Soil | 6.14 | 34 |  |
|  | KSS | Kulik Forest | 25° 38' 13.2" N  88° 7' 4.79" E | Soil | 6.21 | 33 |  |
