# Supplementary material for: Isolation, characterization, identification, genomics and analyses of bioaccumulation and biosorption potential of two arsenic-resistant bacteria obtained from natural environments
Source: Sci Rep. 2024 Mar 8;14:5716. doi: 10.1038/s41598-024-56082-6 (PMC10924095; doi:10.1038/s41598-024-56082-6)
Supplement: Supplementary file 7 — Supplementary Table S3. [file 41598_2024_56082_MOESM7_ESM.docx]

**Supplementary Table S3** Results of different biochemical tests of the potent As tolerant isolates KG1D and PF14.

| **Name of Isolate** | **Catalase test** | **Oxidase test** | **Urease test** | **Gelatin hydrolysis test** | **Lipid hydrolysis test** | **Starch hydrolysis test** | **Citrate Utilization test** | **Methyl red test** | **Indole production test** | **Voges- Proskauer test** |
| --- | --- | --- | --- | --- | --- | --- | --- | --- | --- | --- |
| KG1D | + | - | - | + | + | - | + | + | - | - |
| PF14 | + | + | + | - | - | + | + | - | - | - |

*‘+’ denotes positive whereas ‘-’ denotes negative response to the test*
