# Supplementary material for: Isolation, characterization, identification, genomics and analyses of bioaccumulation and biosorption potential of two arsenic-resistant bacteria obtained from natural environments
Source: Sci Rep. 2024 Mar 8;14:5716. doi: 10.1038/s41598-024-56082-6 (PMC10924095; doi:10.1038/s41598-024-56082-6)
Supplement: Supplementary file 9 — Supplementary Table S5. [file 41598_2024_56082_MOESM9_ESM.docx]

**Supplementary Table S4** List of different heavy metal salts used in the study.

| **Sl. No.** | **Salts of Heavy Metal** | **Formula** | **Manufacturer** |
| --- | --- | --- | --- |
| 1 | Sodium arsenite | NaAsO_2_ | NICE |
| 2 | Sodium arsenate | Na_2_HAsO_4_.7H_2_O | NICE |
| 3 | Copper sulphate | CuSO_4_.5H_2_O | MERCK |
| 4 | Nickel sulphate | NiSO_4_.6H_2_O | SRL |
| 5 | Potassium dichromate | K_2_Cr_2_O_7_ | NICE |
| 6 | Mercuric chloride | HgCl_2_ | NICE |
| 7 | Zinc sulphate | ZnSO_4_.7H_2_O | MERCK |
| 8 | Cadmium chloride | CdCl_2_ | MERCK |
