# Supplementary material for: Isolation, characterization, identification, genomics and analyses of bioaccumulation and biosorption potential of two arsenic-resistant bacteria obtained from natural environments
Source: Sci Rep. 2024 Mar 8;14:5716. doi: 10.1038/s41598-024-56082-6 (PMC10924095; doi:10.1038/s41598-024-56082-6)
Supplement: Supplementary file 10 — Supplementary Legends. [file 41598_2024_56082_MOESM10_ESM.docx]

**Supplementary figure captions**

Fig. S1 Nutrient agar plate showing the colony morphology of pure cultures of the bacterial isolates **(a)** PF14 and **(b)** KG1D.

Fig. S2 Scanning electron micrographs of the As-tolerant isolates **(a)** PF14 and **(c)** KG1D grown in control and in presence of As(III) **(b)** PF14 and **(d)** KG1D.

Fig. S3 Comparative pH tolerance and growth of **(a)** KG1D and **(b)** PF14 at different pH depicted by recording O.D. values after 24 h of growth.

Fig. S4 Combined effect of pH and As(III) on the growth of **(a)** KG1D and **(b)** PF14.

**Supplementary Table captions**

Table S1 The details of isolates along with their MIC values of As(III) and As(V) obtained during this study.

Table S2 Details of the sampling sites including geographical location, pH, temperature and As content of the samples.

Table S3 Results of different biochemical tests of the potent As tolerant isolates KG1D and PF14.

Table S4 The compiled translated BLAST (BLASTX) results of the top two hits for the genes *arsB*, *arsC* and *arsR* of the *ars* operon detected in the genome of the As resistant isolates PF14 and KG1D.

Table S5 List of different heavy metal salts used in the study.
